# The learning environment of paediatric interns in South Africa

**DOI:** 10.1186/s12909-017-1080-3

**Published:** 2017-11-29

**Authors:** Kimesh L. Naidoo, Jacqueline M. Van Wyk, Miriam Adhikari

**Affiliations:** 1 KwaZulu-Natal Department of Health, Department of Paediatrics and Child Health, King Edward VIII Hospital, Nelson R Mandela School of Medicine University of KwaZulu-Natal, Durban, South Africa; 2 Department of Clinical and Professional Practice, Nelson R Mandela School of Medicine, University of KwaZulu-Natal, Durban, South Africa; 3 Department of Paediatrics and Child Health, Nelson R Mandela School of Medicine, University of KwaZulu-Natal, 719 Umbilo Road, Durban, South Africa

**Keywords:** Internship, Medical education, Learning environment, Work-based learning, Graduate, Lower middle income countries (LMIC), Psychometrics, Evaluation studies, South Africa, Postgraduate hospital educational environment measure (PHEEM)

## Abstract

**Background:**

South African (SA) paediatric interns (recently qualified medical graduates) work in a high disease burdened and resource deficient environment for two years, prior to independent practice. Perceptions of this learning environment (LE) influences their approaches to training as well as the outcomes of this period of development. Obstacles to creating a supportive LE and supervisor interaction affects the quality of this training. Measuring perceptions of the LE with validated instruments can help inform improvements in learning during this crucial period of medical education.

**Methods:**

The aims of this study was to determine the psychometric qualities of the Postgraduate Hospital Educational Environment Measure (PHEEM) amongst paediatric interns across four hospital complexes in South Africa and to measure the LE as perceived by both interns and their supervisors. Construct validity was tested using factor analysis and internal consistency was measured with Cronbach’s alpha.

**Results:**

A total of 209 interns and 60 supervisors (69% intern response rate) responded to the questionnaire. The PHEEM was found to be very reliable with an overall Cronbach’s alpha of 0.943 and 0.874 for intern and supervisors respectively. Factor analysis using a 3-factor solution accounted for 42% of the variance with the teaching subscale having the best fit compared with the other sub-scales of the original tool. Most interns perceived the learning environment as being more positive than negative however, their perceptions differed significantly from that of their supervisors. Poor infrastructural support from institutions, excessive workloads and inadequate supervision were factors preventing optimal training of paediatric interns.

**Conclusions:**

The SA version of the PHEEM tool used was found to be a reliable and valid instrument for use in interns amongst high disease burdened contexts. Various obstacles to creating an ideal learning environment for paediatric interns were identified to be in need of urgent review. Key differences in perceptions of an ideal learning environment between interns and their supervisors need to be fully explored as these may result in sub-optimal supervision and mentoring.

**Electronic supplementary material:**

The online version of this article (10.1186/s12909-017-1080-3) contains supplementary material, which is available to authorized users.

## Background

The South African (SA) medical internship program occurs in an environment of high neonatal, infant and child mortality reflecting the multiple disease burdens of HIV/AIDS and Tuberculosis within the poor socio-economic context of sub-Saharan Africa [1–4]. High patient to doctor ratios and challenges with the provision of quality medical education, confound this context for the newly qualified intern [5–7]. Studies of intern training in South Africa reflects high levels of stress [8] and burnout [9–11]. It is in this environment that there is a need to effectively train medical practitioners to care for children.

The learning environment (LE) has been defined as a ‘set of factors that describe a learners’ experience within an organization’ [12]. It has been seen to consist of three parts. The first part entails a physical component which encompasses the provision of food, shelter and comfort, which has been described as being under ‘external regulation’. The second part entails an emotional component including aspects of support, feedback as well as the extent of harassment, which is viewed as a ‘beneficial affective climate’. The third part, an intellectual component, refers to evidence based practice, learning with patients, structured education and instilling motivation which encompasses the ‘learning content and coaching’ [13, 14]. The LE influences trainee’s approaches to learning and the quality of their learning outcomes [15, 16]. Satisfaction with the LE plays a critical role in the success of trainees future achievements [17, 18]. The Postgraduate Hospital Educational Environment Measure (PHEEM) is a well-recognized instrument to assess the learning environment of postgraduate medicine [19]. It has been used internationally, in hospital settings and among interns to assess the learning environments in post-graduate medicine [12, 20–26]. The PHEEM has been shown to have the ability to identify strengths and weaknesses in the LE and scores have a significant negative correlation with burnout levels of those assessed [21]. Structural and cultural differences that exist in the high disease burdened environment of SA may affect the reliability and validity of a tool developed in a very different context. In order for the PHEEM to be used in the SA context it’s psychometric qualities needs to be assessed.

Understanding the learning environment of an educational program is fundamental to managing educational development and change [27]. It is also important to measure the perceptions of the LE amongst both paediatric interns and their supervisors, as both can have very differing perceptions of an ideal LE [17, 28]. This added insight will improve evaluations of the LE. By monitoring and evaluating perceptions of the learning environment, improvements can be made to the quality of training in an informed way.

The aims of this study were to:

- Determine the reliability and validity of the Postgraduate Hospital Educational Environment Measure (PHEEM) as a useful tool to measure the learning environment of interns in SA; and
- Assess the learning environment of interns doing paediatrics in a SA setting and to compare the perceptions of the learning environment between interns and their supervisors.

## Method

### Research design

The study was a cross-sectional cohort study.

### Setting

The SA internship program encompasses a 24-month training period in various specialties including 4 months in paediatrics. Internship in SA is the responsibility of the national government through provincial departments of health using the platform of regional hospitals in each province. The Health Professionals Council of South Africa (HPCSA) is the professional regulatory body is responsible for the oversight and accreditation of curricula, supervisors and the regional hospitals where interns train. Supervisors are usually specialists employed by the regional hospitals and residents working with these specialists. Many supervisors have an affiliation to a medical university however the university structures do not have a formal responsibility in internship training [29].

### Subjects

In order to assess the LE in a high disease burdened context, four major regional hospital complexes in Durban and Pietermaritzburg, in KwaZulu-Natal (KZN) Province, SA were chosen. These hospitals account for 76% of all intern training in SA’s second most populous province where the HIV/TB disease burdens are amongst the highest in the country [30]. Across these four hospital complexes there were 89 senior (specialists) and junior (residents) supervisors who were responsible for the training, mentoring and assessment of interns during the paediatric rotation [30].

Each of the 40 items on the PHEEM questionnaire is scored on a 5-point Likert scale (1 = strongly disagree to 5 = strongly agree). Three subscales related to teaching, role autonomy and social support were proposed by the original developers of the instrument following qualitative and quantitative methods of research [19].

Minor changes to accommodate differences in terminology and for use in South Africa and in a paediatrics specialty were made to the original instrument [19], and it was then piloted with a group of interns at one intern complex and senior intern supervisors across the province in a focus group in order to ensure face validity. The same modified questionnaire used for interns was adapted to use with supervisors to facilitate comparisons across these groups. Additional file 1: Appendix A and Additional file 2: Appendix B reflect the outcomes of this process.

### Procedure

Ethical approval for the study was obtained from the University of KwaZulu-Natal Biomedical Research Ethics Committee and permission granted from the various institutions as well as the Health Research and Knowledge Management Subcomponent of the Department of Health in the province of KZN.

The PHEEM questionnaire was group administered by the primary author to interns on site at each of the hospitals in December 2015. All interns and intern supervisors were informed of the study and invited to participate. Written consent was required for participation. Participants were informed of their rights and could withdraw at any stage. The supervisors completed the questionnaire individually (i.e. a self-administered format). This was done within the same time–period used to survey the interns at each hospital. The questionnaire took approximately 15 min to complete.

### Sample size

The total sample size needed to be representative of the general intern pool in the province as well as greater than 100 for the factor analysis for a 40–item instrument [31]. The achieved sample sizes of 209 interns and 60 supervisors corresponded to a power of 92% when comparing PHEEM scores between two groups using an independent samples t–test for the detection of a medium effect size (Cohen’s d = 0.5) with 80% power at the 5% significance level. Sample size calculations were carried out in G*Power [32].

### Statistical analysis

The original PHEEM questionnaire used a 0–4 scoring range [19] whilst we followed a more conventional 1–5 range as used by some authors [17, 33]. Items 7,8,11 and 13 were reverse-scored.

In order to validate the use of the PHEEM in our setting we studied the psychometric characteristics and internal consistency of the version used in our study. To investigate the internal structure of the PHEEM, especially the construct validity of the original three subscales, we applied factor analysis with varix (orthogonal) rotation to determine the underlying dimensions in the data. The Kaser-Gutmann Eigenvalue criterion of > 1; the Cattell criterion of accepting factors above point of inflexion on the scree plot, and the proportion of the total variance explained (60%) were used to determine the number of underlying factors. Factor loadings above 0.4 were interpreted. Cronbach’s alpha coefficient was used to assess reliability and internal consistency.

Descriptive statistics were calculated of the overall score and that of the three subscales. Continuous variables were summarized by the mean with standard deviation and median with interquartile ranges. The overall PHEEM scale and sub-scale scores were calculated for each participant. Where there was missing data, means were computed based on data for available items, provided this did not exceed 20% of the items. The overall score was computed as the average of all 40 items.

Each item on the PHEEM questionnaire was compared between the interns and the supervisors treating the scores as a continuous measure and comparisons made using the student t–test provided the data met the assumptions for this test. The strength of the associations was measured by the Cohen’s d for parametric tests. As examining the means of the responses may fail to highlight extent of problems elicited especially the perceptions of interns and supervisors on individual items e.g. perceptions of racism, gender bias or a ‘blame culture’ we further categorized each item as ‘strongly disagree’ or ‘other’, in order to determine the extent of difference in these items between interns and supervisors. Data analysis was carried out using SAS Version 9.4 for Windows. The 5% significance level was used throughout (*p*-values < 0.05 indicating significant results).

## Results

### Participants

**Fig. 1:**
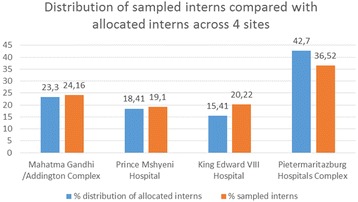
Distribution of sampled interns across five hospital complexes

Two hundred and nine completed questionnaires were returned of 378 interns who had completed paediatrics by the sampling date. Interns perform substantial shift work, and as a result 20% of all eligible interns were not available at the time of the group administration of the survey. The corrected response rate was calculated at 69.2% (209/302 available interns). Figure 1 indicates the distribution of participants from each of the regional hospitals and compares this with the distribution of interns working in each hospital.

Females comprised 55% of the intern sample and all participants were aged 23–37 (mean 26.2, standard deviation 2.6). The response rate of the supervisors was 67% (60/89). The supervisors were 61% female and consisted of 50% senior supervisors (mean age 43 years) and 50% junior supervisors with a mean age of 29 years.

### Factor analysis

**Fig. 2:**
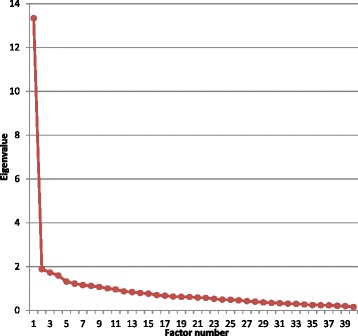
Scree plot of the eigenvalues of the factors Reliability

Factor analysis (FA) was only performed on the data obtained from the group of 209 interns. The FA on all 40 factors suggested ten factors using the Eigenvalue criterion or nine factors (percentage variance explained aiming at 60%) or two or three factors using the inflexion point on the screen plot (See Fig. 2).

With the PHEEM instrument having 40 items in its inventory the scree plot inflexion point is considered an acceptable way to establish the number of factors [28]. The solutions with large number of factors had many factors with fewer than three items with loadings > 0.5.

The two– and three–factor solutions were evaluated and these explained 38% and 42% of the total variance respectively (See Table 1).

**Table 1:**
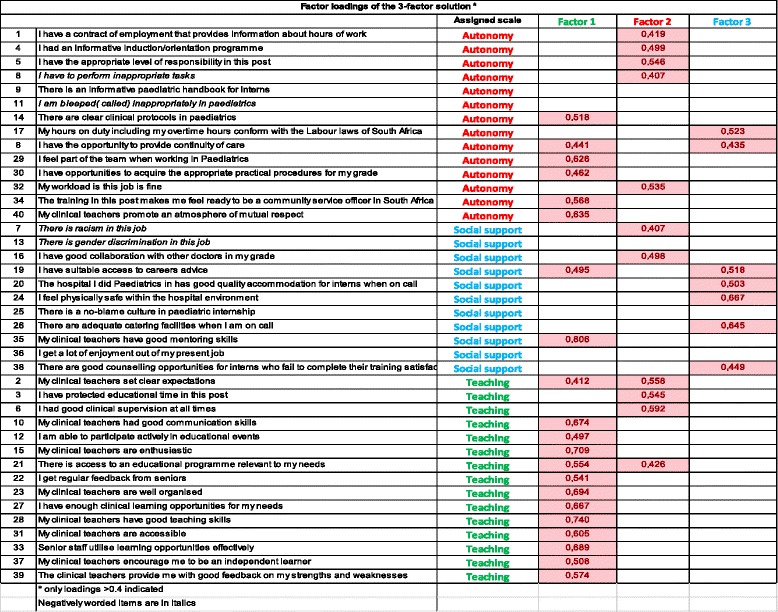
Factor analysis of PHEEM data

A one–factor solution was evaluated and 33% of total variance could be explained by this solution. The three–factor solution had all factors having at least three items each with a loading of > 4. Four items had loadings that were < 0.4. Factor one included the majority of items originally allocated to the teaching subscale (13 out of 21 items). The rest of the items came from the original role autonomy subscale (6 out of 21) and from the social support sub-scale (2 out of 21). The items allocated to factor two belonged to the perceptions of teaching subscale (4 out of 11), social support (2 out of 11) and the largest number from the perceptions of role autonomy (5 out of 11). The third factor included items from the original role autonomy (2 out of 7) and social support subscales (5 out of 7).

### Internal consistency

For the intern group, the Cronbach’s alpha to assess internal consistency was 0,943 for the overall scale. Cronbach’s alpha for the autonomy, teaching and social support subscales are tabulated in Table 2 and were also above 0.7. We ran a Cronbach’s alpha for the supervisor group and the overall Cronbach’s alpha was 0,874. The teaching subscale for both the larger intern group and the supervisor group was above 0,8.

**Table 2:** PHEEM scores with Cronbach’s alpha results for Intern and Supervisor groups

The means, standard deviations, Cronbach’s alpha and *p*-values for the overall and subscale PHEEM scores for interns and their supervisors							
Score type	Interns *n* = 209			Supervisors *n* = 60			*P* –value
	Mean	Standard deviation	Cronbach’s alpha	Mean	Standard deviation	Cronbach’s alpha	
Overall PHEEM score	3.51	0.51	0.943	3.79	0.32	0.874	0.0001
Teaching subscale	3.57	0.6	0.815	3.85	0.4	0.804	0.0007
Role autonomy subscale	3.64	0.48	0.920	3.98	0.34	0.699^a^	<0.001
Social Support Subscale	3.3	0.54	0.760	3.47	0.41	0.675^b^	0.032

^a^0.71 on removal of items 1 and 32

^b^no improvement with removal of any item

### Comparison of intern and supervisor perceptions of the learning environment

Subsequent analysis was based on the original subscales and not those from the factor analysis. The means and the standard deviations were calculated for each item and their overall and subscale means were compared.

**Fig. 3:**
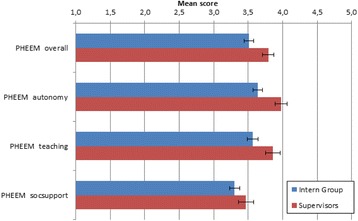
Bar graph showing the mean score scales of the overall PHEEM scale and the three sub-scales of interns and supervisors

The overall and subscale scores were then compared between the interns and their supervisors (See Fig. 3).

There was a significant difference in the overall scores between the interns and the supervisors with the supervisors perceiving the learning environment more positively. The means of the interns sub-scores for the perceptions of teaching and autonomy was significantly lower than their supervisors.

Table 3 illustrates those individual items where the differences between intern and supervisor perceptions were significant.

**Table 3:** Ranking of key items where interns and supervisors significantly differ in perceptions

PHEEM item	Key items where the differences between interns and supervisors was significant^a^ *(based on % Interns who disagree* ^b^ *with PHEEM item statement “highest to lowest”)*	% Disagree: interns	% Disagree: supervisors	*p*-value	Subscale
17	My hours on duty including my overtime hours conform with the Labour laws of South Africa	50,2	7,0	< 0.0001	Autonomy
25	There is a no-blame culture in paediatric internship	39,0	22,0	0,020	Social support
32	My workload is this job is fine	33,8	15,3	0,0059	Autonomy
22	I get regular feedback from seniors	26,8	12,3	0,023	Teaching
7	*There is racism in this job*	26,2	5,1	0,0002	Social support
8	I have to perform inappropriate tasks	24,0	3,5	0,0002	Autonomy
6	I had good clinical supervision at all times	21,5	6,8	0,012	Teaching
18	I have the opportunity to provide continuity of care	21,2	7,0	0,012	Autonomy
21	There is access to an educational programme relevant to my needs	18,3	7,0	0,041	Teaching
13	*There is gender discrimination in this job*	17,0	3,5	0,0087	Social support
40	My clinical teachers promote an atmosphere of mutual respect	16,1	5,1	0,031	Autonomy
11	*I am bleeped(called) inappropriately in paediatrics*	15,6	1,8	0.00029	Autonomy

^a^with all other items the difference between interns and supervisors who disagree(*or agree in reverse scored items*) with statements did not reach statistical significance

^b^ *or agree with reverse scored item*

The key items accounting for differences between interns and supervisors in the Teaching sub-scale related to lack of feedback from seniors, clinical supervision, and access to appropriate educational programmes.

The key items accounting for differences between interns and supervisors in the Autonomy sub-scale related to perceptions of overtime hours done, amount of workload, performing inappropriate tasks, lack of continuity of care, and lack of mutual respect. The key items accounting for differences between interns and supervisors in the Social Support sub-scale related to lack of a ‘no-blame’ culture, presence of racism and gender discrimination.

## Discussion

The PHEEM instrument was validated within a high disease burdened context in SA with significant differences being noted in how interns and their supervisors perceived the LE in this context. The interns sampled adequately reflected their distribution across various hospitals and the response rate was in keeping with similar surveys that utilized PHEEM elsewhere [22, 23, 26].

The modified PHEEM used established a good internal consistency as reflected by a high Cronbach’s alpha value. The overall reliability was good particularly for the teaching subscale across both intern and supervisors’ surveys and the high value was similar to that found in other studies [28, 34]. The high Cronbach’s alpha however suggests that one underlying construct seen as the ‘overall educational environment’ is being reliably measured using the modified PHEEM in this setting [17, 22].

### Construct validity

When comparing the factor analysis performed to the existing scales of the original instrument, the correspondence was not a clear fit. The teaching subscale performed much better than the other two scales. Whilst there was less of a clear fit with the original role autonomy and social support sub-scales, the second factor dealt mainly with the contractual and governance aspects of internship, orientation, contracts on work hours, type of tasks and responsibility. This seemed to corroborate with the original role autonomy scale of the original instrument. The third factor related loosely for support of the intern indicated by items relating to accommodation, safety, career advice, and support of ‘at risk’ interns. This factor can be seen to corroborate with the original subscale on social support. Some studies indicate the uni-dimensionality of the PHEEM scale [17, 34, 35] whilst others support its multi-dimensionality [22, 28]. In a demographically divergent intern group in SA, across different hospitals, the PHEEM did not clearly perform as a multidimensional tool. Further enquiry into how these individual characteristics of interns may affect differing perceptions of the LE and the use of the PHEEM is needed in the SA context.

The use of the PHEEM tool with the three original subscales has been noted to be convenient for summarising and comparing results [22, 28]. Using the interpretation proposed by the developers of the original PHEEM, interns across the four sampled hospital complexes perceived the LE in paediatrics as more positive than negative however with ‘room for improvement’ being noted [19]. This finding is similar to evaluations conducted internationally [22, 26, 28, 33, 36–38]. This finding also resonates with various studies in other provinces in SA, indicating reasonable adequacy in how internship prepares interns for later practice with significant challenges still being noted [39–42].

### Issues to be addressed

The three major challenges noted included issues related to infrastructure and institutional management; work-load issues and issues relating to the quality of supervision. Institutional infrastructural challenges for interns related mainly to poor catering and accommodation. Whilst international PHEEM evaluations from well-resourced countries highlighted similar issues with catering and accommodation [36, 43], in the SA context this challenge in institutional management has been associated with the overall poor governance of public hospitals. This issue has been shown to greatly add to demotivation among interns [44, 45].

Over a third of the intern respondents indicated that they felt the workload, working hours and type of tasks as excessive or inappropriate for interns in paediatrics. An excessive workload and work hours posed major challenges to SA interns and has been shown to infringe on labor laws [46]. Excessive work hours and workload are well documented as major contributors of high levels of stress and burnout in junior doctors in SA [8–10]. Adherence to existing legislative frameworks need to be applied urgently to ensure that excessive work hours do not compromise the safety, health and occupational functioning of interns or patients. Oversight by accreditation bodies is required to ensure that these frameworks are adhered to. The third cause for concern in the teaching and learning environment is related to the adequacy of mentoring and supervision during internship. More than a quarter of interns indicated the presence of a ‘blame culture’ in paediatrics and insufficient feedback especially for ‘at risk’ interns. A lack of dedicated education time, inadequate guidance and career advice was also noted. An additional concern, noted by nearly a quarter of interns, related to a perceived culture of racism and gender discrimination in the LE. This is of significance noting the rapid changes in the demographic composition of SA’s newly qualified interns.

Of further concern however was the differences in perceptions on many of these issues between interns and supervisors. The significant differences found between the overall PHEEM and subscales scores relating to teaching and role autonomy between interns and their supervisors clearly points to this mismatch.

While supervisors recognize deficiencies in infrastructural challenges that concur with intern’s perceptions, aspects related to teaching and working, seem to differ. A clear understanding of roles and responsibilities needs to be identified and consensus must be developed between interns and supervisors related to working and learning. This process needs to commence at orientation programs and through the training period for both interns and supervisors. It is likely that perceptions of supervision are influenced by the experiences of busy, inadequately trained and poorly motivated supervisors [47]. The disconnect between interns and their supervisors reflect sub-optimal supervision with poor communication, inadequate mentoring with lack of quality assessment practices being in place. Our findings corroborate that of various other studies on internship in SA which call into question the quality and quantity of direct supervision and on-going assessment by adequately trained staff [47, 48]. Improving supervision and assessment has been noted to be a major factor that can improve internship in S.A [43]. These issues of’ work versus learning and the attitude of supervisors and their expected roles as ‘evaluators and coaches’ have been highlighted as the major tensions of internship [49]. In SA this underlying tension must also be contextualized in the rapidly changing demography of recently qualified medical students in comparison with the supervisor cohort and this need to be examined further as a potential reason for this schism [50]. Discrepancies between the supervisors’ and interns’ perceptions of the learning environment could also be explained by the difference concerning the views of trainers and trainees of the ideal training environment [17, 28]. Understanding the expectations of interns in achieving expected competencies and the role of assessment towards these ends, needs to be defined and evaluated in the SA context. The discrepant perceptions among supervisors and interns of the same LE indicates a need to improve our understanding of the ‘community of practice’ (COP) within the internship setting [51]. Further research and more qualitative insight into this ‘community of practice’ during internship will likely improve our understanding and is required especially in high disease burdened and resource constrained contexts.

The dissemination of regular evaluations of the LE using validated, standardized tools such as the PHEEM, to the accrediting bodies (HPCSA), health departments and directly to intern supervisors to ensure informed feedback can occur can serve as a means monitoring, comparing and improving the training of interns across hospitals and disciplines.

### Limitations

While the PHEEM was developed mainly to assess the educational environment of postgraduate students in hospital settings that is especially residents, various authors have reported on its use with interns [22–26]. It is unlikely that the factor structure would differ substantially between interns and postgraduates given the similarity of the workplace [14].

The interns were sampled only in one province and the study was not replicated in other provinces to compare how a moderate or inadequate supply of resources impacted on the LE. We are however confident that the KZN province closely represents the South African situation with its high disease burden and resource poor regional public hospitals.

The intern response rate was 69% and could have been higher if further sampling occurred with interns who were on leave or had to attend to on call duties at the time of the survey.

Our assessment focused only on the domain of paediatrics but it did so across different hospital complexes and across two cities in KZN. Significant differences in the educational environment between different specialties and hospitals have been noted possibly indicating the importance of the general climate at the training hospital and the internal climate within each department as having a significant effect on the quality of the educational environment [24].

## Conclusion

This study demonstrated that the PHEEM had good internal consistency and thus serves as a valid tool to assess the learning environment of interns in a high disease-burdened context like KZN, SA.

While paediatric interns in KZN perceived the learning environment as satisfactory, significant obstacles were observed in the development of an ideal learning environment. Poor infrastructural support at institutional level, excessive patient loads, excessive work hours and sub-optimal supervisor interaction for mentoring and ongoing assessment impacted on the notion of an ideal learning environment. These factors have been implicated as major contributors for high stress and burnout among interns in SA and need to be urgently reviewed. The significant differences observed in the perceptions of the learning environment between interns and their supervisor’s requires further insight into this relationship.

## Additional files

Additional file 1: Appendix A: Modified PHEEM for Interns in South Africa. (XLSX 11 kb)

Additional file 2: Appendix B: Modified PHEEM for Intern-superviors in South Africa. (XLSX 11 kb)
